# Severe Case of Idiopathic Chronic Pericarditis Treated by Video Transthoracic Pericardial Fenestration

**DOI:** 10.7759/cureus.93486

**Published:** 2025-09-29

**Authors:** Yuko Harada, Tomoya Uchimuro

**Affiliations:** 1 Cardiology, Kawasaki Municipal Ida Hospital, Kawasaki, JPN; 2 Internal Medicine, Harada Naika Clinic, Kawasaki, JPN; 3 Cardiovascular Surgery, Kawasaki Saiwai Hospital, Kawasaki, JPN

**Keywords:** cardiogenic cerebral embolism, chronic pericarditis, idiopathic recurrent pericarditis, thoracoscopic left atrial appendage clipping, video transthoracic pericardial fenestration

## Abstract

Idiopathic chronic pericarditis is rare and difficult to treat. Most cases are benign and self-limited; however, a small number of cases develop severe symptoms and could be fatal. They may be refractory to medication and pericardiocentesis. We report a case of severe pericarditis in an 83-year-old patient who presented with systemic edema, weight gain of 15 kg over 2 years, jaundice, and shortness of breath. Chest CT scan and echocardiogram revealed a massive pericardial effusion, which suggested chronic pericarditis. Edema was resolved with diuretics, but pericardial effusion remained. Pericardiocentesis was performed, and the etiology was unknown. The patient developed an ischemic stroke, and the treatment for recurrent pericarditis became very difficult. Thoracoscopic left atrial appendage (LAA) clipping and pericardial fenestration were performed simultaneously. The patient was free from recurrence for 8 weeks. Such recurrent pericarditis with severe symptoms that require surgical treatment is rare. Transthoracic pericardial fenestration could be an optimal treatment for such high-risk patients.

## Introduction

Pericarditis is the inflammation of the pericardium, which may cause fluid congestion in the pericardial sac. Acute pericarditis is an inflammatory pericardial syndrome with or without pericardial effusion. Recurrent pericarditis is diagnosed with a documented first episode of acute pericarditis, a symptom-free interval of 4-6 weeks or longer, and evidence of subsequent recurrence of pericarditis [1,2]. When the symptoms of pericarditis last more than 3 months, it is called chronic pericarditis.

Chronic pericarditis is caused by cancer, viral infection, tuberculosis, collagen diseases, trauma, radiation, or cardiac surgery. Idiopathic or recurrent pericarditis is usually less symptomatic. The treatments are pericardiocentesis, nonsteroidal anti-inflammatory drugs (NSAIDs), steroids, or colchicine [1,2]. As idiopathic pericarditis usually develops in senior patients over months or years with minimal symptoms, invasive treatment is not always required. Severe cases with massive pericardial congestion are rarely seen. They are often refractory to medication and pericardiocentesis.

We experienced a severe case of idiopathic chronic pericarditis, which developed severe systemic edema, congestive liver, and jaundice. These symptoms were refractory to pericardiocentesis. The patient developed a stroke triggered by diuretics. Therefore, transthoracic pericardial fenestration was performed.

## Case presentation

An 83-year-old man presented with systemic edema and progressive shortness of breath. On examination, the patient was hemodynamically stable and required no oxygen to maintain saturation at rest. His blood pressure was 120/75 mmHg, heart rate was 70 bpm, and oxygen saturation was 97%. The face, the body trunk, the arms, and the legs were significantly swollen. Patient gained body weight of 15 kg over 2 years, and more than 5 kg during the preceding month. Exudates were oozing out from the numerous cracks on the legs. Past history included hypertension, type 2 diabetes, and brainstem hemorrhage.

**Figure 1:**
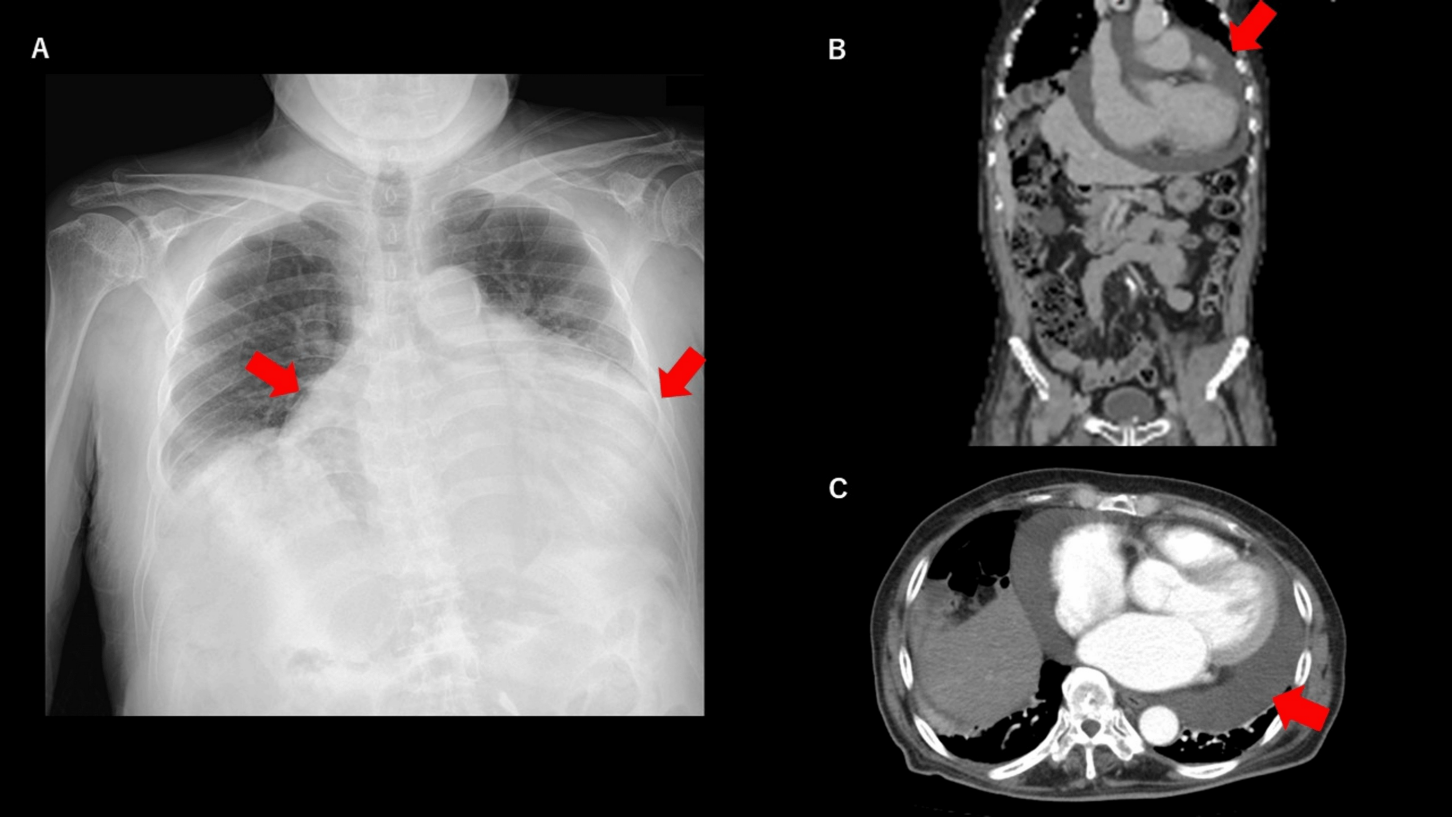
Chest X-ray and CT scan before surgery. A) chest X-ray before surgery. Significant cardiomegaly is shown with red arrows. B) sagittal view of contrast body CT scan. Pericardial effusion is remarkable (red arrow). C: axial view of contrast chest CT scan. Significant cardiomegaly and pericardial effusion are shown (red arrow).

Chest X-ray revealed remarkable cardiomegaly with CTR 80%. EKG revealed atrial fibrillation (AF). Whole body CT scan revealed systemic subcutaneous edema, especially in the lower extremities, cardiomegaly, pericardial effusion, bilateral pleural effusion, and dilated inferior vena cava (IVC) as shown in Figure 1.

Blood test results were as follows: WBC 7100/μl, Hb 10.9 g/dl, PLT 157 × 103/μl, Protein 5.8 g/dl, Alb 3.6 g/dl, BUN 19.5 mg/dl, Cr 0.83 mg/dl, T.B. 1.2 mg/dl, AST 24 IU/l, ALT 26 IU/l, LDH 297 IU/l, glucose 194 mg/dl, CRP 2.76 mg/dl, CEA 5.0 ng/ml, CA19-9 negative, anti-nuclear antigen negative. Urine protein was negative; therefore, nephrotic syndrome was ruled out.

Echocardiography also revealed a remarkable pericardial effusion that was congested along the entire circumference of the heart. Ejection fraction (EF) was 55% and the other parameters were within normal limits except for a mildly dilated left atrium. Diastolic collapse of the right ventricle (RV) was not observed; therefore, cardiac tamponade was not likely. As the previous CT scan of 2 years ago also revealed a remarkable pericardial effusion, the patient was diagnosed with chronic pericarditis.

Furosemide 40 mg/day was administered intravenously. Systemic edema started to resolve; however, leg edema and pericardial effusion remained, and jaundice appeared with an elevated TB level of 1.9 mg/dl. On the 14th day after admission, the patient underwent pericardiocentesis. Approximately 800 ml of yellow-colored, clear fluid was drained. The laboratory test results of the fluid were as follows: pH 7.8, cell count 775/μl (mononuclear 94%, polynuclear 6%), protein 4.1 g/dl, glucose 200 mg/dl, LDH 213 IU/l, albumin 2.93 g/dl, ADA 22.3 IU/l, CEA 2.2 ng/ml, and CA19-9 negative. The pericardial fluid was transudative, and cytology revealed only small numbers of lymphocytes. The cause of pericarditis was considered to be idiopathic.

After pericardiocentesis, the bilirubin level became normal, and leg edema was also resolved. The patient was discharged from the hospital; however, cardiomegaly recurred 4 weeks after pericardiocentesis. Oral furosemide was continued to prevent edema. As the HASBLED score was 4 (hypertension, history of hemorrhagic stroke, anemia, and elderly), the dose of edoxaban was decreased to 15 mg/day.

Three weeks after discharge from the hospital, the patient developed a brain stroke. Mechanical thrombectomy was performed for an acute ischemic stroke of the right middle cerebral artery (MCA). The anticoagulation therapy was strengthened to apixaban 10 mg/day. Dehydration by furosemide and low-dose non-vitamin K antagonist oral anticoagulant (NOAC) was considered to be the cause of cardiac stroke.

Echocardiography revealed a relapse of massive pericardial effusion. As the patient was at high risk of stroke and bleeding, repetitive pericardiocentesis was not recommended. After a detailed discussion, the patient and his family chose to undergo radical treatment.

**Figure 2:**
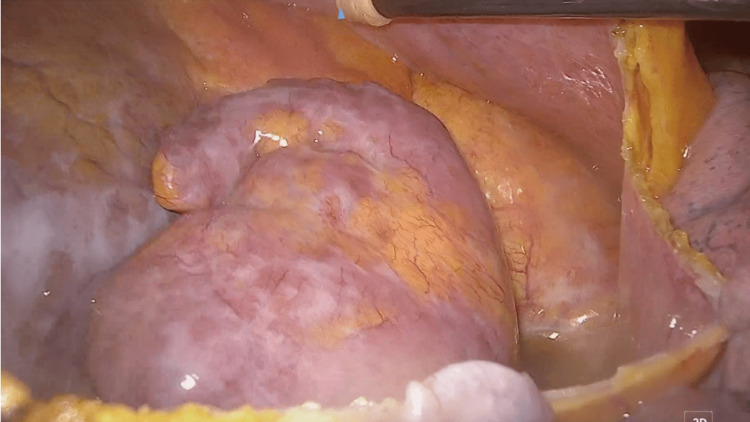
The Left atrial appendage is exposed. After incising the pericardium, the left atrial appendage was exposed.

**Figure 3:**
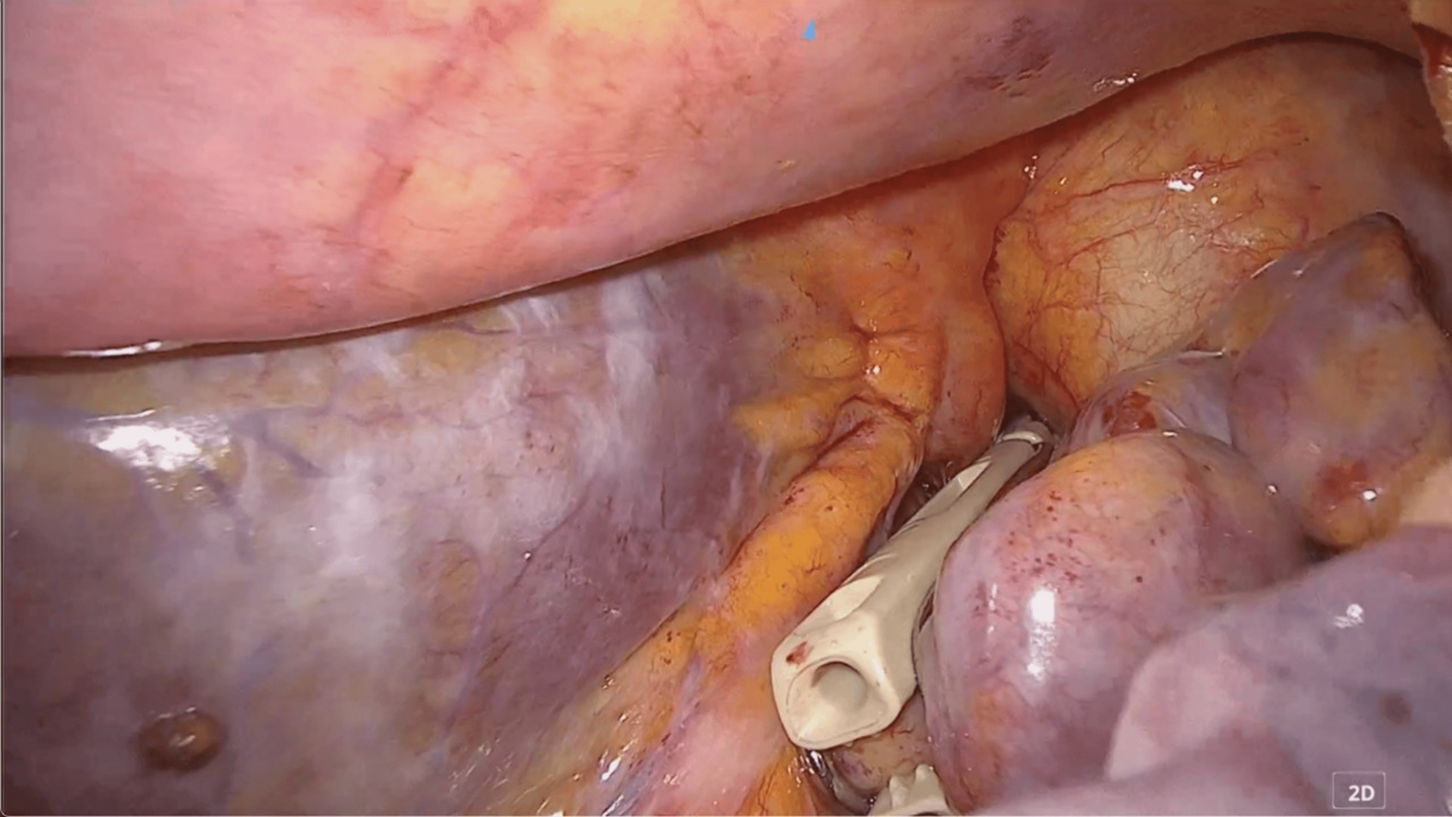
The Left atrial appendage was clipped. The left atrial appendage was closed using a device.

Thoracoscopic LAA clipping and pericardial fenestration were performed simultaneously. Pulmonary vein isolation or maze procedure was not considered due to the patient’s long history of AF. The main port was placed in the left 6th intercostal space, and the left lung was deflated by CO2. The assisting port was placed in the 4th intercostal space, and the camera port was placed in the 5th intercostal space. The pericardium was incised at 2 cm dorsal to the phrenic nerve. Incision was made toward the reflection of the pericardium (central end) and toward 2 cm caudal of LAA (peripheral end). LAA was sized, and a 50mm (Medtronic, Minneapolis, USA) was selected for LAA clipping. Using Penditure, the LAA was clipped to close as shown in Figure 2-3.

**Figure 4:**
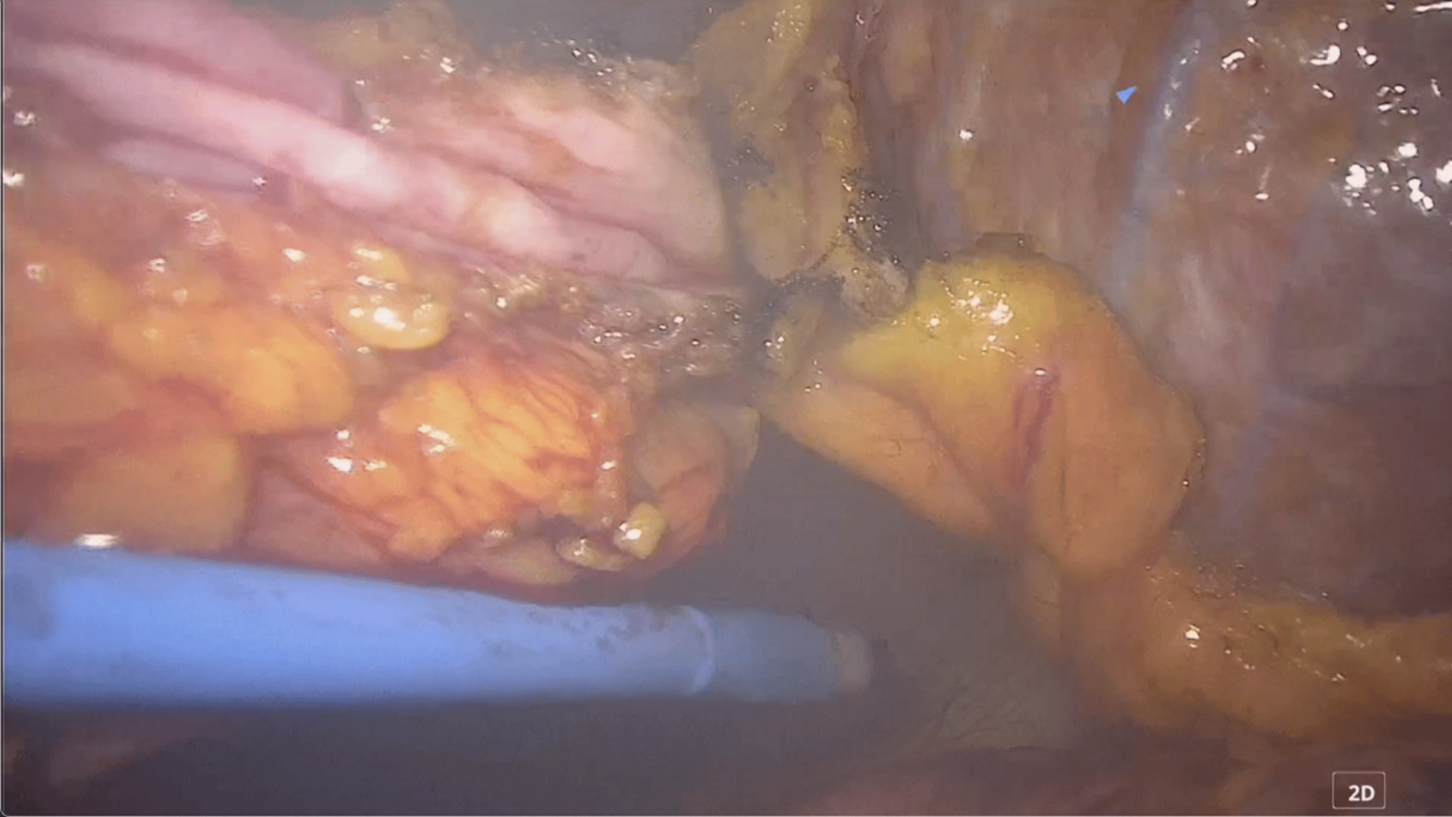
Resection of the pericardium. Anterior pericardium, ventral to the phrenic nerve, was resected.

**Figure 5:**
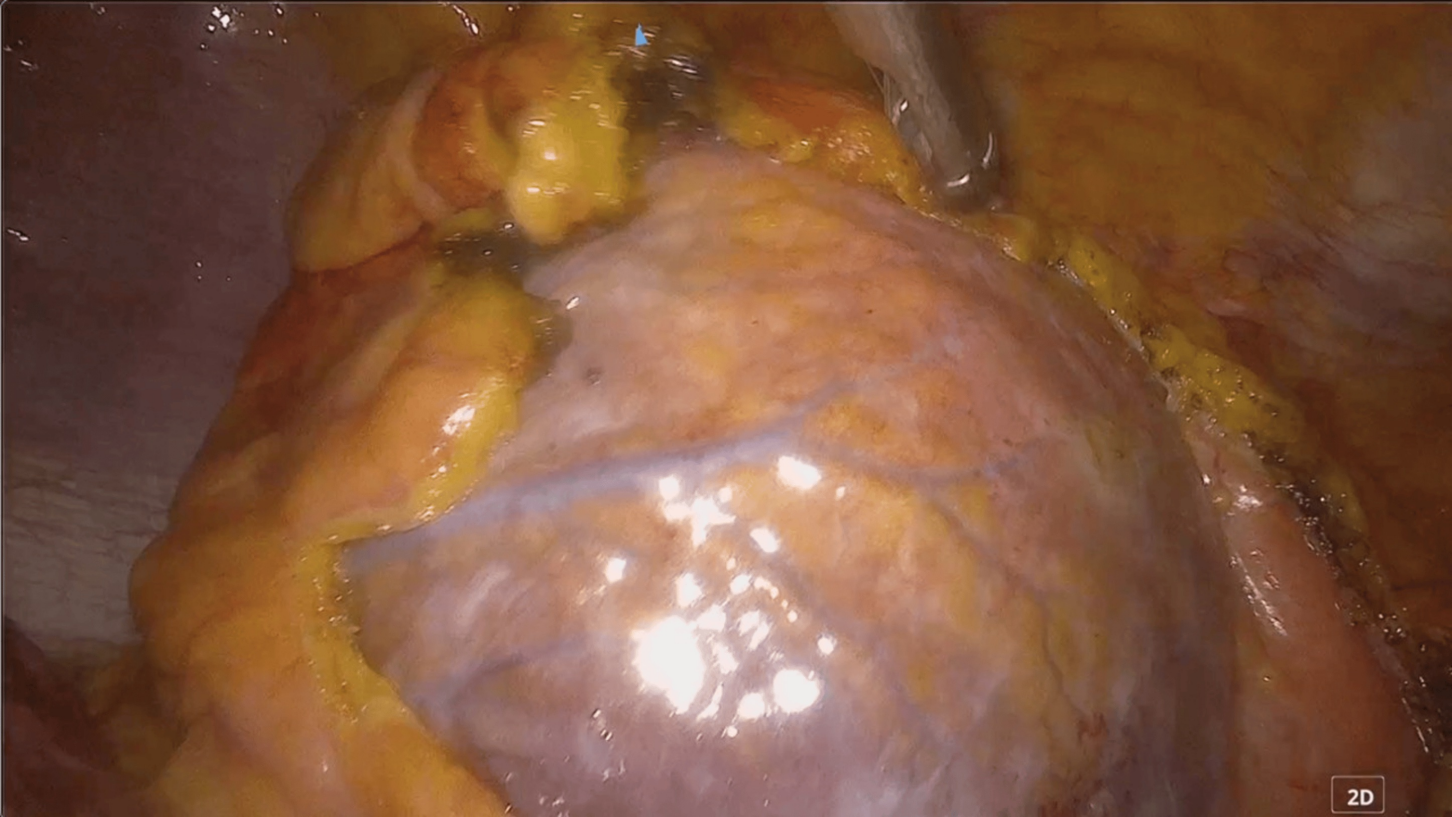
Pericardial fenestration was performed. A wide pericardial window was created.

The anterior pericardium was resected about 8 cm × 5 cm in order to create a large “window” to drain the pericardial fluid into the left thoracic cavity. The window is as shown in Figures 4-5.

**Figure 6:**
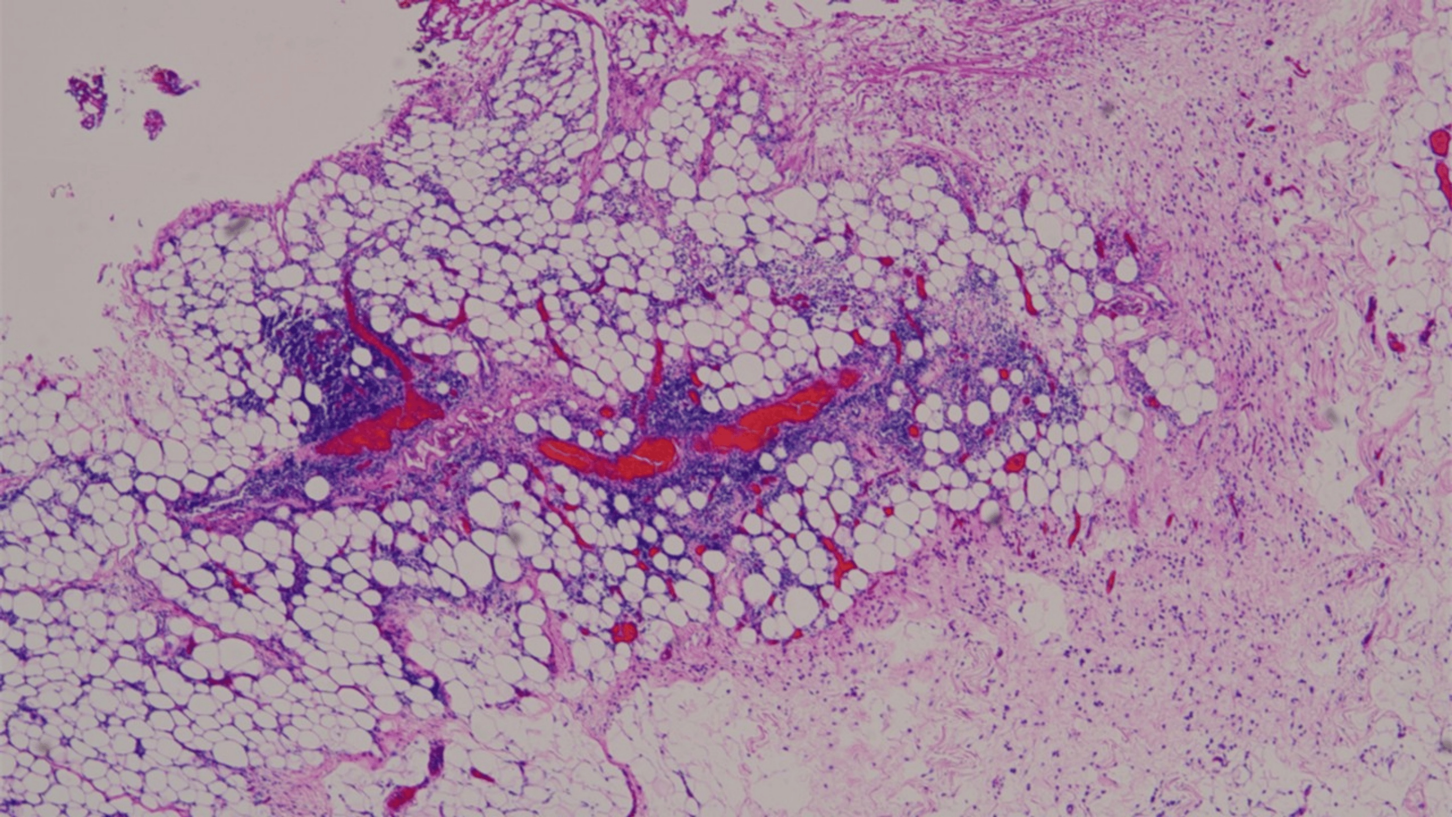
Histopathological image of pericardium, stained with hematoxylin and eosin. Mesothelial cells are observed on the surface of the pericardium, and the wall is supported by collagen fibers. Focal clusters of lymphocytes and plasma cells are shown. These findings are consistent with a mildly inflamed pericardium.

A Blake drain 24 Fr was placed in the left thoracic cavity. The total time of surgery was 1 hour and 53 minutes. Pathological examination of the pericardium revealed mild inflammation with lymphocytes and plasma cells. A histopathological image of the pericardium, stained with hematoxylin and eosin, is shown in Figure 6.

**Figure 7:**
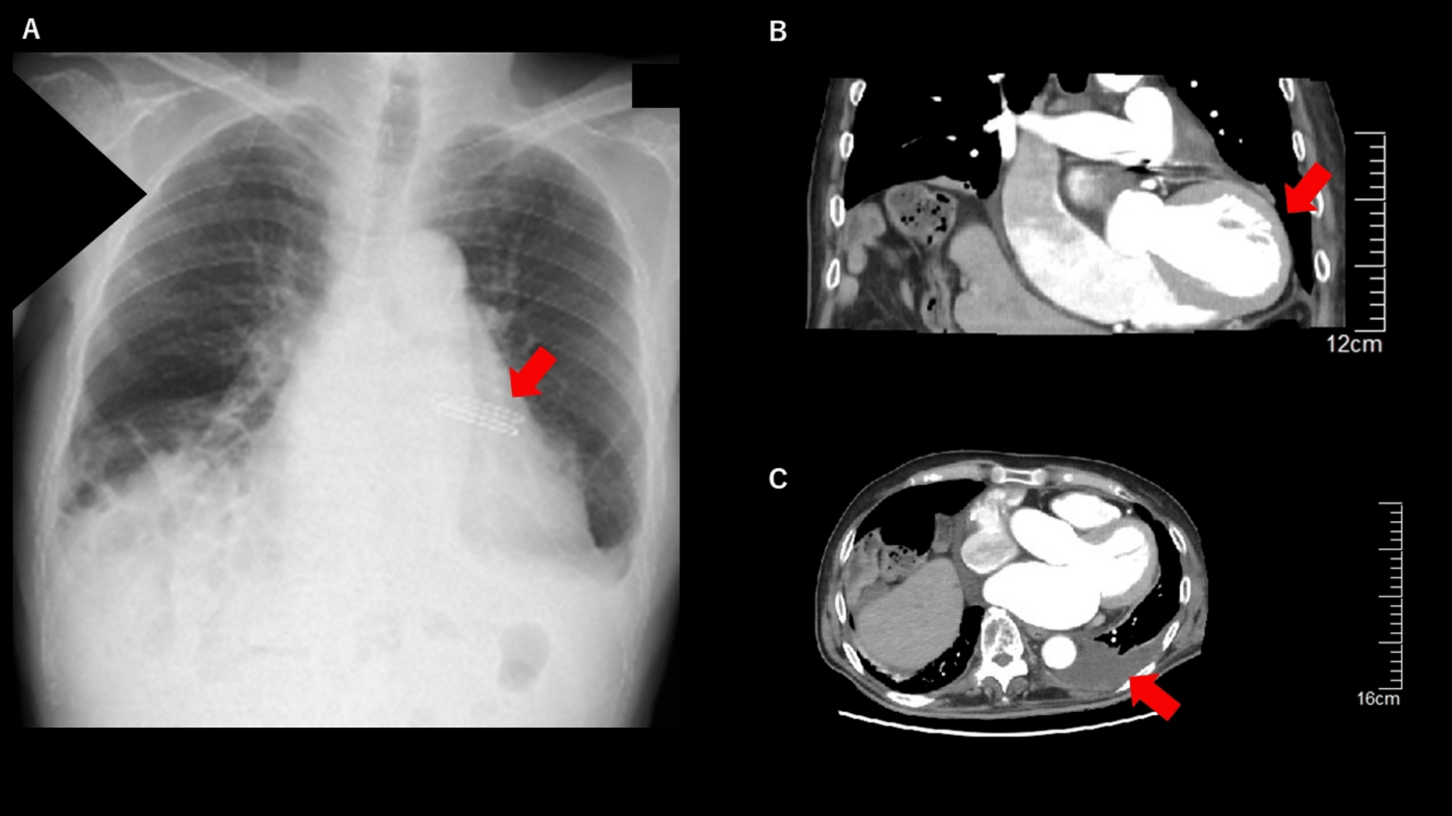
Chest X-ray and contrast CT scan after surgery. A) chest X-ray after surgery. The LAA clipping device is shown (red arrow). B) Sagittal view of contrast chest CT scan after surgery. Pericardial effusion remarkably decreased (red arrow). C) axial view of contrast CT scan after surgery. A small amount of pleural congestion is observed (red arrow).

There were no complications after surgery. The Blake drain was removed 3 days post-surgery. Colchicine 1 mg/day was initiated to prevent recurrence of pericarditis. Eight weeks post-surgery, the patient remained free of adverse symptoms. There was no relapse of pericardial fluid congestion, jaundice, or edema. The most recent chest X-ray and chest CT are shown in Figure 7.

## Discussion

Acute pericarditis is overall benign and self-limiting [2]. The etiology of acute pericarditis is primarily infectious (80-85%), such as viral infection and tuberculosis [3]. Other etiologies are autoimmune diseases (up to 10%), neoplastic diseases (5-7%), metabolic diseases such as uremia or myxedema, and drugs [3]. After the first episode of acute pericarditis, the rate of first recurrence is reported at 15-30% of cases [4]. A minority of patients (5-10 %) revealed additional recurrence within 3 months, developing into chronic pericarditis [3,4].

The present case has shown massive pericardial effusion for over 2 years with unknown etiology. Uremia, myxedema, autoimmune diseases, trauma, and cancer were all ruled out. The symptoms progressed slowly over 2 years; however, at hospital admission, the patient presented with critical symptoms of right heart failure. Cardiac tamponade and constrictive carditis were less likely with echocardiogram and CT scan findings. However, pericardiocentesis was necessary for life-saving. Even though most cases of idiopathic chronic pericarditis are benign and asymptomatic, the present case was critical.

According to the 2015 European Society of Cardiology (ESC) guidelines, the main indication (for pericardial window) is represented by recurrent large effusions or cardiac tamponade when a more complex operation, such as pericardiectomy, is a high risk or the life expectancy of the patient is reduced (e.g., neoplastic pericardial disease) and the intervention is palliative [2]. The surgical indication for the present case was discussed with the patient, his family, and cardiosurgeons. Patient has a past history of brainstem hemorrhage and a recent cardio-embolic stroke in addition to diabetes, AF, hypertension, and old age. Since he was at very high risk of recurrence of stroke due to invasive treatment, a video transthoracic surgery was scheduled 7 weeks after the incident of stroke.

Transthoracic surgery for pericarditis was originally performed for cardiac tamponade after cardiac surgery [5,6]. It is performed for high-risk patients who cannot tolerate another open-heart surgery. It is said that a small incision window of 1-2 cm may cause relapse, so that a larger window is recommended [7]. The present case underwent surgery to create a large window in the pericardium and had no relapse afterwards. ESC guidelines recommend using NSAIDs or colchicine for acute and recurrent pericarditis [2]. As the patient has asthma, colchicine was selected.

The treatment strategy of pericardial fenestration and colchicine is thus very effective for high-risk elderly patients with idiopathic chronic pericarditis. Severe cases are rare but could be fatal. Thoracoscopic pericardial fenestration should be considered for such cases.

## Conclusions

Severe cases of idiopathic chronic pericarditis are rare and difficult to treat. Pericardial effusion is often recurrent and resistant to medication. Surgical treatment is difficult for high-risk patients; however, a video transthoracic pericardial fenestration can be safely performed with no recurrence. The treatment strategy of transthoracic surgery followed by colchicine could therefore be more positively considered for high-risk patients with severe symptoms.
